# Unveiling the shared genes between systemic sclerosis and lung cancer

**DOI:** 10.3389/fmed.2024.1431642

**Published:** 2024-12-18

**Authors:** Pengfei Pan, Xin Liu, Yun Wang, Huixuan Wang, Cheng Xu, Junhui Lu

**Affiliations:** ^1^Department of Rheumatology and Immunology, The Affiliated Huai’an Hospital of Xuzhou Medical University, The Second People’s Hospital of Huai’an, Huai’an, China; ^2^Department of Dermatology, The Affiliated Huai’an Hospital of Xuzhou Medical University, The Second People’s Hospital of Huai’an, Huai’an, China

**Keywords:** systemic sclerosis, lung cancer, differentially expressed genes, ferroptosis, PRKG2

## Abstract

The risk of lung cancer is significantly increased in patients with systemic sclerosis (SSc), yet the specific genes underlying this association remain unexplored. Our study aims to identify genes shared by SSc and lung cancer. We identified differentially expressed genes (DEGs) from SSc and lung adenocarcinoma (LUAD) datasets (SSc: GSE95065, LUAD: GSE136043) in the GEO database. We found shared genes by intersecting top genes in protein–protein interaction networks by the STRING database. The area under the ROC curve (AUC) was calculated for each shared gene in validation datasets (SSc: GSE231692; LUAD: GSE43458), identifying PRKG2 as the core shared gene. We used the UALCAN platform to assess PRKG2 expression in LUAD patients at various stages and lymph node metastasis states, and compared disease-free survival (DFS) between low and high PRKG2 expression LUAD groups. PRKG2 was overexpressed in A549 cells to study its impact on lung cancer cell proliferation and invasion *in vitro*. We identified seven shared genes (SCN7A, AGTR1, WIF1, PRKG2, LTF, AQP4, COL10A1), with the AUC for PRKG2 exceeding 0.93 in both diseases (SSc AUC = 0.973; LUAD AUC = 0.939). The PRKG2 expression levels of LUAD patients with different clinical stages and lymph node metastasis states were consistently lower than those observed in normal individuals. The DFS of LUAD patients in the high PRKG2 expression group was higher than that in the low expression group (*p* = 0.028). *In vitro* experiments confirmed elevated PRKG2 expression inhibits the proliferation and invasion of lung cancer cells. PRKG2 is one of the genes shared by SSc and lung cancer, affecting the proliferation and invasion of lung cancer cells.

## 1 Introduction

Systemic sclerosis (SSc) is a complex disease characterized by small vessel lesions, autoantibody production, and fibrosis affecting the skin and visceral organs (1). The incidence rate of SSc is 1.4 cases per 100,000 person-years (2). This disease significantly diminishes patients’ quality of life and life expectancy (3–5). Interstitial lung disease, pulmonary arterial hypertension, infections, and tumors are common causes of mortality in SSc patients (5).

Compared to the general population, SSc patients exhibit a significantly increased risk of tumorigenesis (6). The incidence rate of tumors in SSc patients varies from 3 to 10% across different populations (6–12). Among SSc patients, lung cancer is the most prevalent tumor, followed by breast cancer (6, 8, 9, 13). Risk factors for the occurrence of lung cancer in SSc patients include long-term lung involvement, smoking, prolonged SSc duration, low age at SSc diagnosis, anti-topoisomerase I antibodies, a history of renal crisis, and male gender (14). Several studies have investigated potential common mechanisms between the two diseases, such as telomere shortening, glycolysis, oxidative stress, microbiome involvement, miRNAs, and LncRNAs (14). However, the relationship between SSc and neoplasms is complex, and there is no consensus among researchers regarding their common mechanisms. Further clarification of the shared mechanisms between these two diseases is necessary.

The occurrence of cancer in SSc patients complicates their therapeutic management. These patients require long-term, sustained immunosuppressive therapy (1), which contrasts with the reliance of anti-tumor therapy on an active immune response. Therefore, investigating potential targets for the prevention and treatment of SSc complicated by lung cancer is crucial. Genes are recognized as key elements in the pathogenesis of diseases. Identifying susceptibility genes could aid in the early prevention and intervention of SSc with concurrent lung cancer. Nevertheless, knowledge about core genes that are common to both SSc and lung cancer is currently limited, highlighting the need for further research.

The rapid advancement of bioinformatics and the availability of public databases have greatly facilitated the identification of pathogenic genes (15). To date, no bioinformatics-based research has explored the core shared genes between SSc and lung cancer. In this study, we employed bioinformatics methods to analyze the core shared genes between SSc and lung cancer, with the aim of identifying potential pathogenic genes and elucidating shared mechanisms.

## 2 Materials and methods

### 2.1 Data source

The data of gene expression profile for SSc and lung cancer were obtained from the Gene Expression Omnibus (GEO) database. The GEO database, maintained by the National Center for Biotechnology Information (NCBI), is a public repository for gene expression data, encompassing both microarray and next-generation sequencing studies. We utilized the GEO database to download and analyze datasets relevant to our research objectives, thereby gaining insights into the molecular mechanisms underlying diseases. Following screening, GSE95065 dataset was selected as the SSc expression dataset, and GSE136043 dataset as the lung adenocarcinoma (LUAD) dataset. The GSE95065 dataset includes transcriptomic sequencing data of skin biopsies from 18 SSc patients and 15 healthy controls (16). The GSE136043 dataset comprises mRNA expression profiles from five primary LUAD tissues and five non-neoplastic tissues (17). Additionally, we utilized the GSE231692 and GSE43458 datasets for validation. The GSE231692 dataset comprises expression profiles from skin biopsies of 33 SSc patients and 14 healthy controls (18). The GSE43458 dataset encompasses gene expression profiles from 80 LUAD samples and 30 corresponding normal lung tissue samples (19). We also utilized the GSE40839 and GSE231693 datasets to analyze the expression levels of the core shared gene in lung tissue fibroblasts of SSc associated interstitial lung disease (SSc-ILD) and normal populations. The GSE40839 dataset includes the expression profiles of fibroblasts in lung tissue from 10 normal controls and 8 SSc-ILD patients (20). The GSE231693 dataset contains expression profile data of fibroblasts from 20 normal lung tissues and 20 SSc-ILD lung tissues (18).

### 2.2 Data standardization and processing

GEO2R, provided by NCBI, is an online tool designed for analyzing gene expression data within the GEO database. The box plots generated by GEO2R were used to display the distribution of expression levels across all samples, which allowed for the assessment of dataset quality, such as variability between samples and the normality of the data. Therefore, we employed GEO2R to confirm the standardization of the GSE95065 and GSE136043 datasets. Differential gene expression analysis was conducted on these two datasets. We applied the “limma” package (version 3.40.6) in R to identify differentially expressed genes (DEGs) (21). Thresholds for differential gene expression were set as *p* < 0.001 and |fold change| > 2 for the LUAD group, and *p* < 0.001 and |fold change| > 1.3 for the SSc group. Subsequently, we utilized Cytoscape software for Gene Ontology (GO) and Kyoto Encyclopedia of Genes and Genomes (KEGG) enrichment analyses on the DEGs (22) and constructed protein–protein interaction (PPI) networks using the STRING database (23), in order to consolidate all available and predicted protein–protein interactions.

### 2.3 Identification of shared genes in SSc and LUAD

We imported both PPI networks into Cytoscape software and screened these networks for top genes using the “cytoHubba” plugin (24), which ranks genes based on their network topological properties. Next, we intersected the top DEGs from both diseases to identify shared genes potentially involved in the pathogenesis of both diseases.

### 2.4 Identifying the core shared gene of SSc and LUAD

Ferroptosis exacerbates pulmonary fibrosis in SSc and leads to cell death in lung cancer, playing a significant role in the pathogenesis and progression of both diseases. Therefore, to identify the core shared gene between the two diseases, we investigated the correlation between these shared genes and ferroptosis-marker genes. All ferroptosis marker genes were sourced from the FerrDb database, the first global repository dedicated to the study of ferroptosis regulators and ferroptosis-disease relevance (25). We employed the “corrplot” R package to evaluate the correlation between the marker genes and the shared genes across the datasets. Furthermore, to determine the discriminatory ability of these shared genes in distinguishing LUAD and SSc patients from normal individuals, we used the “pROC” R package to construct receiver operating characteristic (ROC) curves and calculate the area under the curve (AUC) for each gene within both the SSc and LUAD validation datasets (26).

### 2.5 PRKG2 and its related genes

We identified gene groups closely associated with PRKG2 using the GeneMANIA database (27). Subsequently, we performed GO and KEGG enrichment analyses on PRKG2 and its associated genes. This analysis aids in elucidating the potential molecular mechanisms underlying PRKG2.

### 2.6 The differential expression, clinicopathological analysis and prognosis of PRKG2 in LUAD

UALCAN is a powerful bioinformatics platform that incorporates sequencing data from the TCGA database (28). Utilizing these data, we assessed the expression levels of PRKG2 and its correlation with cancer staging and lymph node metastasis status in LUAD patients. GEPIA2 is a comprehensive platform designed for personalized analysis, offering data from 9,736 tumor types and 8,587 normal samples within the TCGA and GTEx projects (29). We employed GEPIA2 to analyze differences in overall survival (OS) and disease-free survival (DFS) among LUAD patients stratified by low and high PRKG2 expression.

### 2.7 Estimation of immune infiltration in LUAD

CIBERSORT, the most frequently cited tool for assessing immune cell infiltration, operates on the principle of linear support vector regression for deconvolution analysis of the expression matrix of human immune cell subtypes (30). We utilized CIBERSORT to evaluate the influence of PRKG2 on immune cell infiltration within lung cancer tissues, with parameters set to signature genes (LM22), batch correction mode (B-mode), and 1,000 permutations. To bolster the credibility of our findings, we also referenced the TIMER database (31), an alternative approach for quantifying immune cell infiltration, to assess the impact of PRKG2 expression on the infiltration levels of distinct immune cell types in LUAD.

### 2.8 Cell culture and transfection

The human-derived LUAD A549 cell line was cultured in 100-mm cell culture dishes at a concentration of 1 × 10^6^ cells/mL for this study. We then added 10 mL of RPMI 1640 complete medium containing 1% Penicillin/Streptomycin solution and 10% fetal bovine serum. When the cell density reached approximately 70% confluence, the PRKG2 overexpression plasmid (FLAG-PRKG2) and the negative control plasmid (FLAG-NC) were transfected into A549 cells using Lipofectamine™ 3000 (L3000075, Invitrogen™, United States) and Opti-MEM (31985, Gibco, United States). After 48 h, three groups of cells (FLAG-PRKG2 overexpression, FLAG-NC control, and blank control) were collected.

### 2.9 Quantitative real time PCR

We extracted total RNA from each group of A549 cells. Subsequently, cDNA was synthesized using a reverse transcription kit. The mRNA expression of PRKG2 was quantified by qPCR using a kit (11143ES50, Yeasen Biotechnology, China), with GAPDH serving as an internal reference gene. The primer sequences were as follows:

PRKG2

forward: 5′-GGTTCCGTGAAACCCAAACA-3′

reverse: 5′-CACCACATCCTGAAGCTTGTT-3′

GAPDH

forward: 5′-ATCATCAGCAATGCCTCCTG-3′

reverse: 5′-ATGGACTGTGGTCATGAGTC-3′.

### 2.10 Cell proliferation experiment

We utilized the Cell Counting Kit-8 (CCK-8, C6005, NCM Biotech, China) to assess the proliferation of A549 cells in both the FLAG-PRKG2 overexpression and control groups. Cells from each group were plated into 96-well plates. After the addition of the CCK-8 reagent, all plates were incubated at 37°C for 4 h, and then the absorbance at 450 nm was measured using a microplate reader.

### 2.11 Wound healing assay

Twenty four-well plates were utilized to culture each group of A549 cells. Upon reaching 100% confluence, a 200-μl pipette tip was used to create a scratch line in each group. The cells were rinsed with PBS, and photographs of the scratches were taken using a microscope. Additional photographs of the two groups of cells were captured after a 24-h interval and compared with the initial images.

### 2.12 Cell migration and invasion assay

Matrigel was applied to the upper surface of Transwell chambers (catalog number 3470, Corning, United States) for the cell invasion assay. Serum-free medium, used to culture each group of A549 cells, filled the upper chamber, whereas the lower chamber contained medium with 20% fetal bovine serum. After a 36-h incubation in a cell incubator, the upper chamber was removed, and the non-invading cells on the upper surface were wiped clean. The cells that invaded through the Matrigel were stained with methylene blue and then visualized and counted under a microscope. Except for the Matrigel application, the remaining steps of the cell migration experiment closely resembled those of the invasion assay. The detailed procedures for cell invasion and migration assays can be referred to the study by Wen et al. (32).

### 2.13 Statistical analysis

We utilized R software (version 4.0.5) for generating all figures. Data from the two groups were compared using an unpaired t-test. The criterion for statistical significance was a *p*-value of less than 0.05. Each assay was repeated a minimum of three times.

## 3 Results

### 3.1 DEGs and functional enrichment analysis

**Figure 1:**
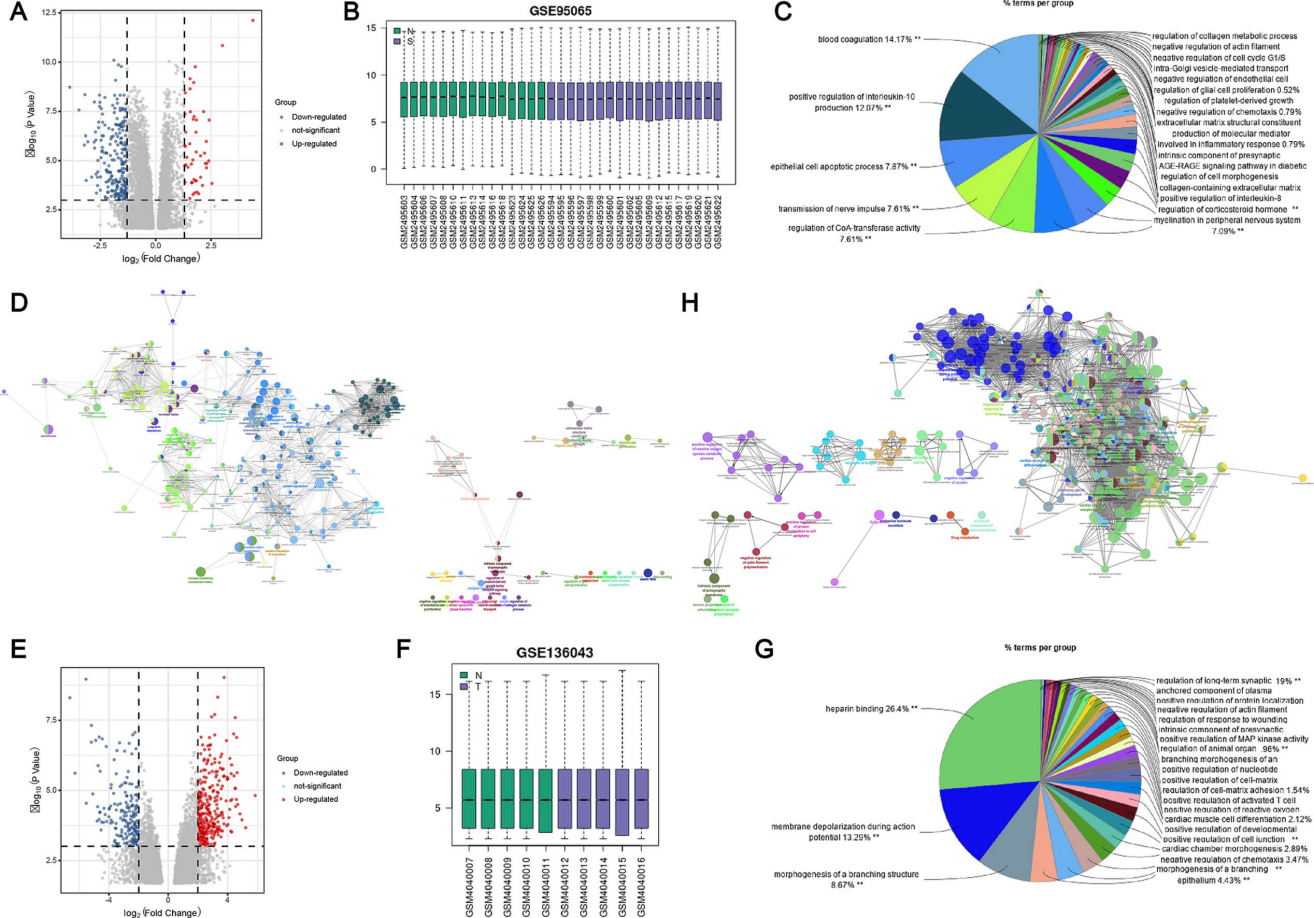
Identification and enrichment analysis of DEGs in SSc and lung cancer datasets. **(A)** Volcano plot of DEGs in the GSE95065 dataset; **(B)** Standardization of the GSE95065 dataset; **(C)** GO functional enrichment analysis of DEGs in the GSE95065 dataset; **(D)** KEGG pathway enrichment analysis of DEGs in the GSE95065 dataset; **(E)** Volcano plot of DEGs in the GSE136043 dataset; **(F)** Standardization of the GSE136043 dataset; **(G)** GO functional enrichment analysis of DEGs in the GSE136043 dataset; **(H)** KEGG pathway enrichment analysis of DEGs in the GSE136043 dataset.

The GEO2R analysis results indicate that the GSE95065 and GSE136043 datasets have been standardized (Figures 1B,F). DEGs are depicted on volcano plots, where upregulated genes are marked with red dots, and downregulated genes with blue (Figures 1A,E). In comparison to normal individuals, the SSc group exhibited upregulation of 47 genes and downregulation of 216 genes. Likewise, the lung cancer group showed upregulation of 274 genes and downregulation of 182 genes. Subsequently, we conducted GO/KEGG functional enrichment analysis for these DEGs. The DEGs from the SSc dataset are associated with various signaling pathways, including blood coagulation, positive regulation of interleukin-10 production, epithelial cell apoptosis, neural impulse transmission, and regulation of CoA-transferase activity. Similarly, The DEGs from the lung cancer dataset are involved in pathways such as heparin binding, membrane depolarization during action potentials, negative regulation of chemotaxis, and morphogenesis of branching structures (Figures 1C,D,G,H).

### 3.2 Shared genes in SSc and lung cancer

**Figure 2:**
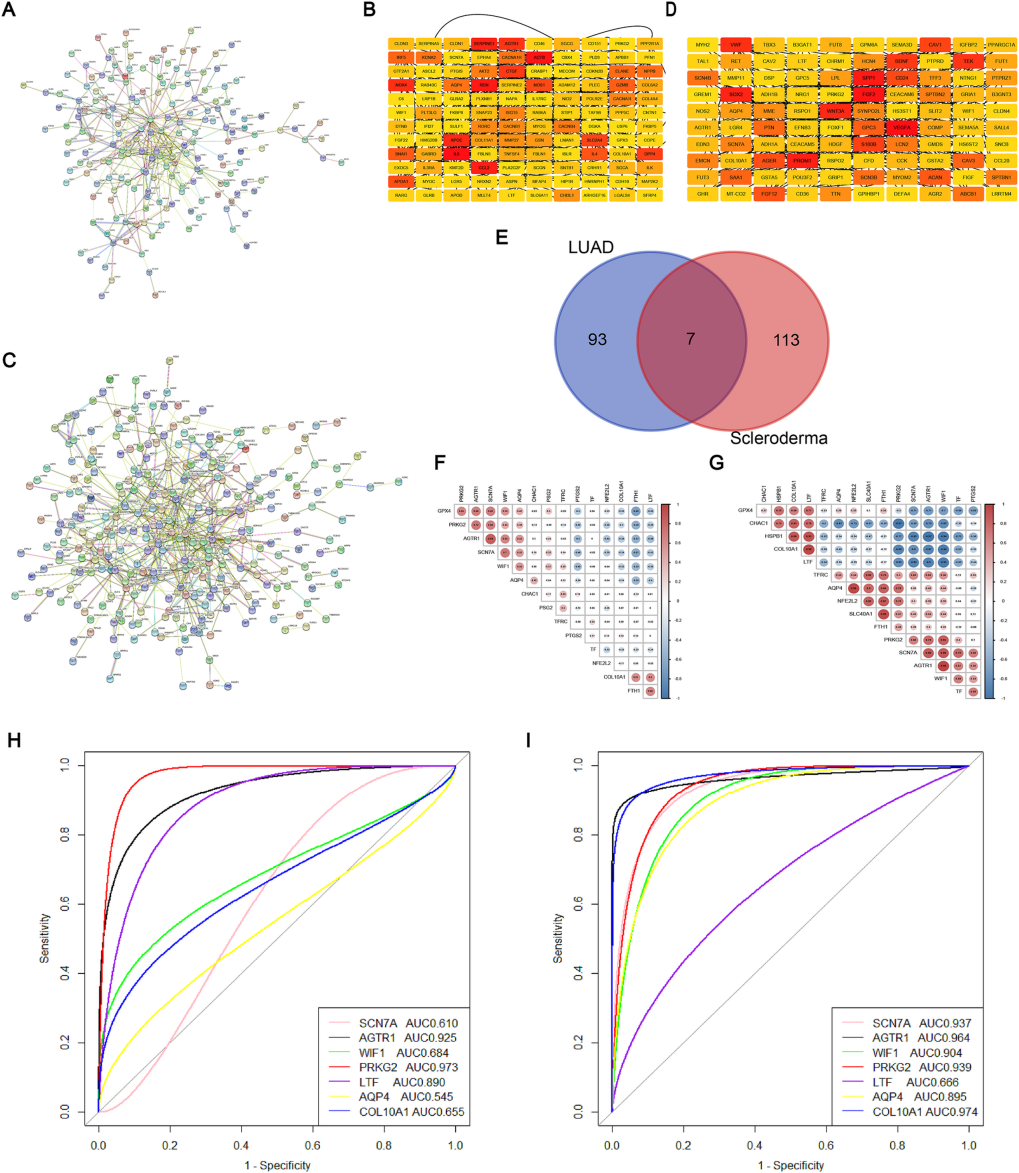
Core shared DEGs from SSc and LUAD datasets. **(A)** PPI network of DEGs from the SSc dataset; **(B)** Heatmap of connections for the top 120 DEGs within the SSc dataset; **(C)** PPI network of DEGs from the LUAD dataset; **(D)** Heatmap of connections for the top 100 DEGs within the LUAD dataset; **(E)** Venn diagram showing the intersection of core DEGs between the SSc and LUAD datasets; **(F)** Correlation heatmap for the seven shared genes and ferroptosis marker genes in SSc; The numbers enclosed in circles refer to the results of the correlation analysis between two genes. **(G)** Correlation heatmap for the seven shared genes and ferroptosis marker genes in LUAD; The numbers enclosed in circles refer to the results of the correlation analysis between two genes. **(H)** ROC curve and AUC for the seven shared genes within the SSc validation dataset; **(I)** ROC curve and AUC for the seven shared genes within the LUAD validation dataset.

We constructed PPI networks for DEGs using the STRING database (Figures 2A,C). Subsequently, we identified the top 100 DEGs from the LUAD dataset and the top 120 DEGs from the SSc dataset. Utilizing the cytoHubba plugin, we identified several DEGs in central positions, including SERPINE1, PRKG2, AGTR1, IL-6, CCL2, and ACTB in the SSc dataset, and PROM1, FGF2, VEGFA, SPP1, PRKG2, and BDNF in the LUAD dataset (Figures 2B,D). By intersecting these top DEGs, we recognized seven genes potentially involved in the pathogenesis of both SSc and lung cancer: SCN7A, AGTR1, WIF1, PRKG2, LTF, AQP4, and COL10A1 (Figure 2E). By analyzing the correlation of these shared DEGs with ferroptosis marker genes, we found that in the SSc group, PRKG2 was significantly positively correlated with GPX4 and negatively with PTGS2, NFE2L2, and FTH1 (Figure 2F). In the lung cancer group, PRKG2 exhibited significant positive correlation with NFE2L2, SLC40A1, TFRC, and significant negative correlation with CHAC1 and HSPB1 (Figure 2G). Additionally, using independent validation datasets (LUAD: GSE43458; SSc: GSE231692), we evaluated the capacity of these shared DEGs to distinguish between the two diseases and the normal population. The results showed that PRKG2 had an AUC greater than 0.93 in both diseases (SSc AUC = 0.973; LUAD AUC = 0.939) (Figures 2H,I). In conclusion, we propose PRKG2 as a core shared gene between SSc and lung cancer.

### 3.3 PRKG2 expression and biological process analysis

#### 3.3.1 Expression levels of PRKG2 in SSc, LUAD, and SSc-ILD group

**Figure 3:**
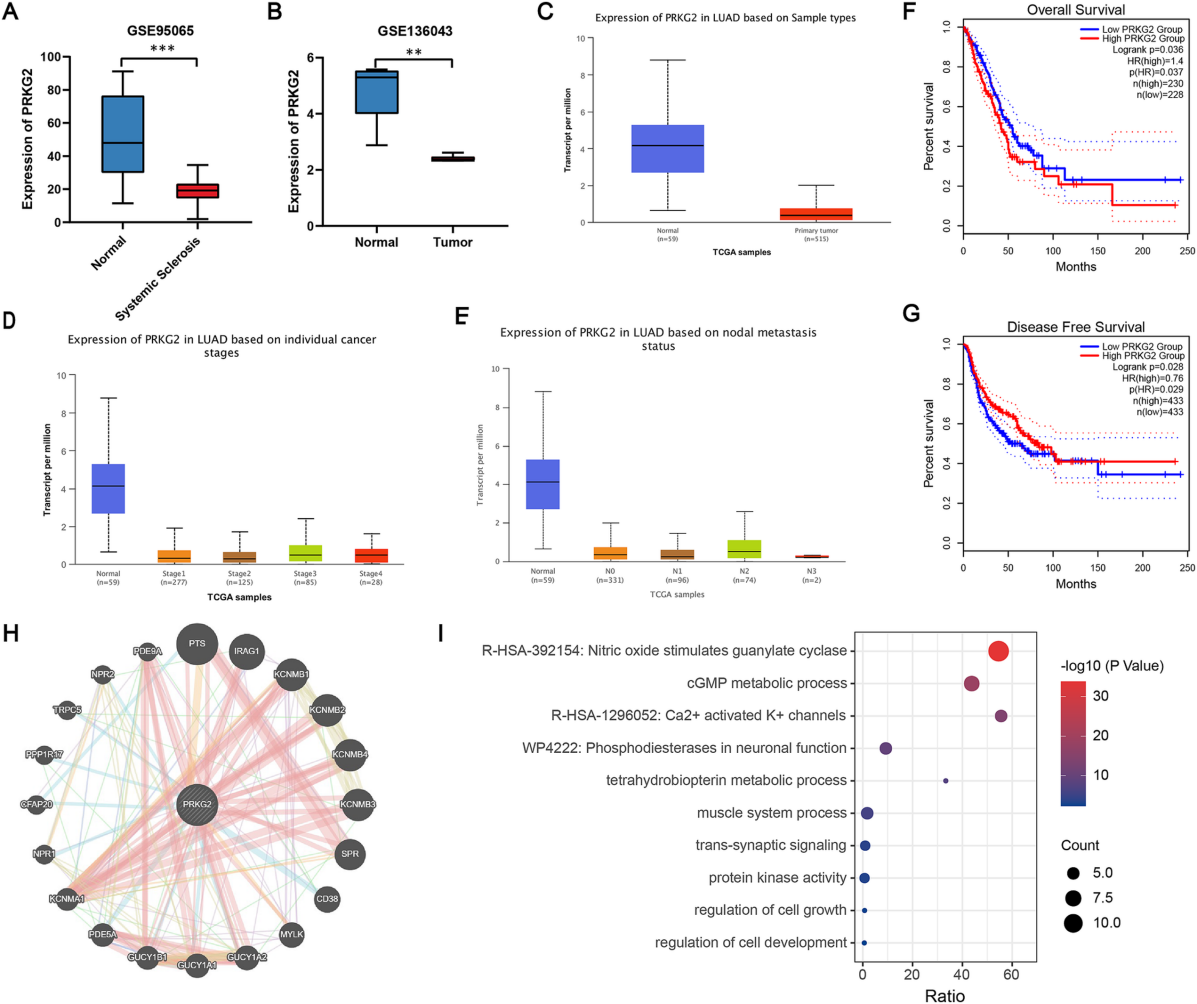
PRKG2 expression levels in tissues and the biological processes associated with PRKG2. **(A)** Expression levels of PRKG2 in the SSc dataset; **(B)** Expression levels of PRKG2 in the LUAD dataset; **(C)** Expression of PRKG2 in LUAD samples as per the TCGA database; **(D)** Expression of PRKG2 in LUAD samples across different pathological stages; **(E)** Expression of PRKG2 in LUAD samples categorized by nodal metastasis status; **(F)** Kaplan–Meier curve for overall survival for low and high PRKG2 expression groups in LUAD; **(G)** Kaplan–Meier curve for disease-free survival of low and high PRKG2 expression groups in LUAD; **(H)** PRKG2 and its associated genes; **(I)** GO/KEGG enrichment analysis for PRKG2 and its associated genes. *** indicates *p* < 0.001; ** indicates *p* < 0.01.

**Figure 4:**
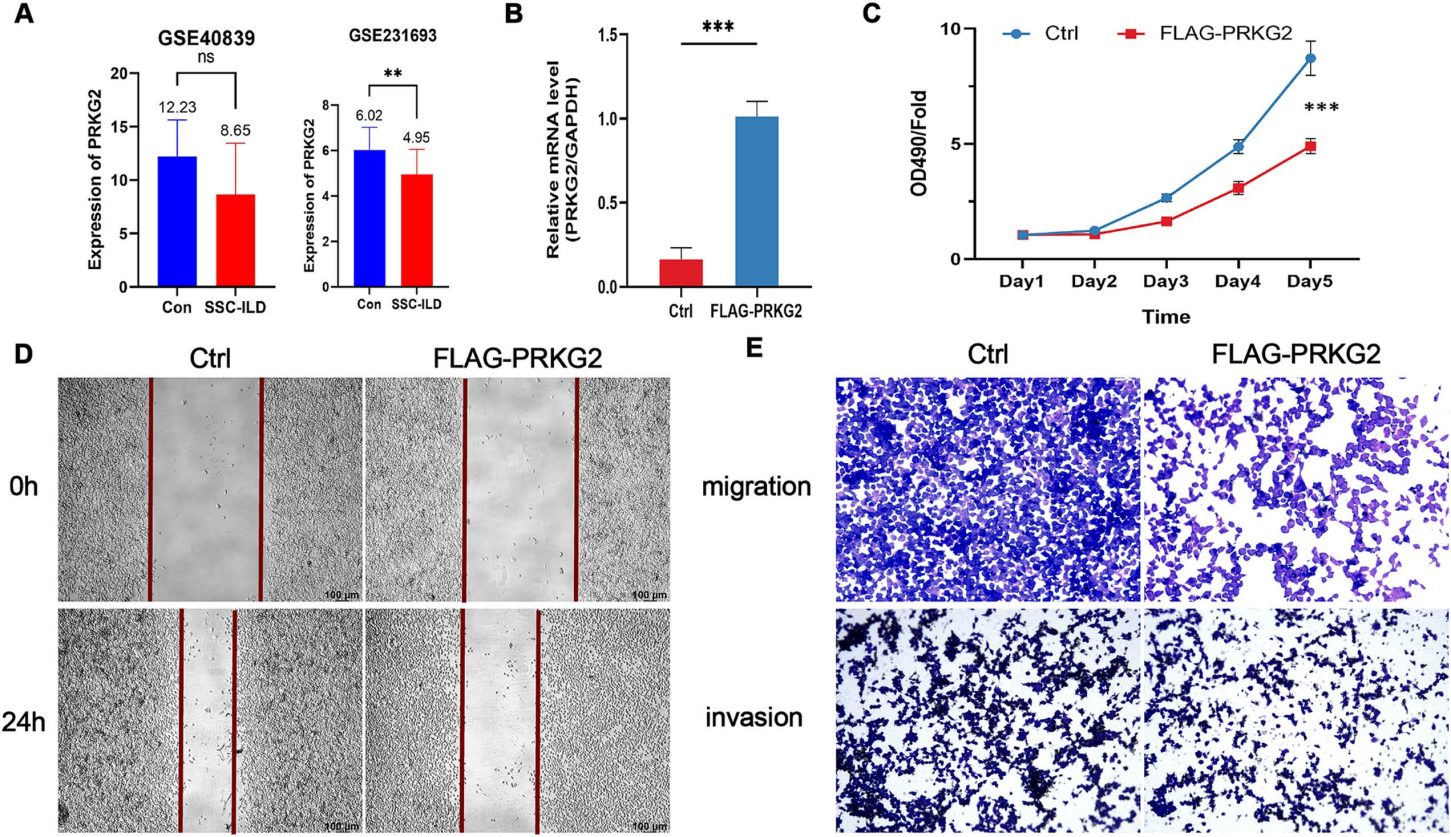
Inhibitory effects of PRKG2 on lung cancer cells *in vitro*. **(A)** Expression levels of PRKG2 in fibroblasts of lung tissues of SSc-ILD and normal populations within the datasets GSE40839 and GSE231693; **(B)** Overexpression of PRKG2 in A549 cells; **(C)** MTT assay for the impact of PRKG2 on A549 cell proliferation; **(D)** Wound-healing assay for the effect of PRKG2 on A549 cell migration; **(E)** Transwell assay for the influence of PRKG2 on A549 cell migration and invasion. *** indicates *p* < 0.001.

The PRKG2 expression levels in tissues from SSc and lung cancer patients were significantly lower than those of normal individuals, as evidenced by the GSE95065 and GSE136043 datasets (Figures 3A,B). Analysis of the TCGA database indicated that primary LUAD patients had lower PRKG2 expression levels than normal individuals (Figure 3C). The expression levels of PRKG2 in pulmonary fibroblasts from lung tissues of SSc-ILD patients were significantly lower than those in fibroblasts from normal lung tissues in the GSE231693 dataset. Additionally, the PRKG2 expression levels in pulmonary fibroblasts from SSc-ILD patients were also lower than in the control group in the GSE40839 dataset, yet this difference was not statistically significant (Figure 4A).

#### 3.3.2 PRKG2 expression and clinical outcomes in LUAD patients

The expression of PRKG2 in LUAD patients with different clinical stages and lymph node metastasis status was significantly lower than that in the normal population (Figures 3D,E). We categorized all lung cancer patients from the GEPIA2 database into low and high PRKG2 expression groups based on the median expression value of PRKG2. The median DFS was significantly longer in the high PRKG2 expression group compared with the low expression group (*p* = 0.028), whereas the median OS was shorter (*p* = 0.037) (Figures 3F,G).

#### 3.3.3 Genes closely related to PRKG2 and its potential signaling pathways involved

To investigate the signaling pathways associated with PRKG2, we identified a close association between PRKG2 and genes such as PTS, IRAG1, KCNMB1, KCNMB4, KCNMB3, SPR, GUCY1A1, and PDE5A (Figure 3H). The GO/KEGG functional enrichment analysis for PRKG2 and these related genes revealed that these genes influence various cellular components, including those involved in the nitric oxide stimulation of guanylate cyclase, cGMP metabolic processes, and calcium-activated potassium channels (Figure 3I).

### 3.4 Correlation of PRKG2 expression with immune cell infiltration in lung cancer

**Figure 5:**
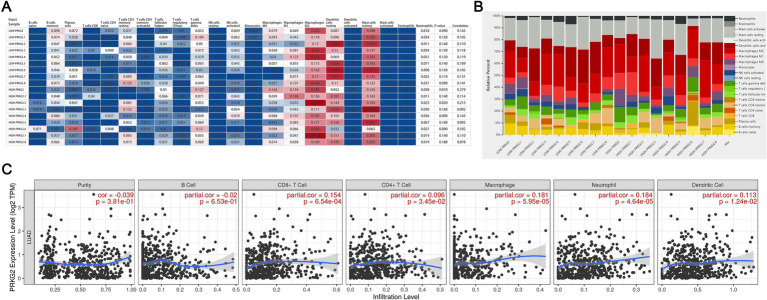
Associations between PRKG2 expression and immune cell infiltration in lung cancer. **(A)** Heatmap depicting the associations between PRKG2 and immune cells; **(B)** Relative percentages of associations between PRKG2 and immune cells; **(C)** Correlations between PRKG2 expression and the infiltration of immune cells.

After analyzing data from the CIBERSORT database, we observed a positive correlation between PRKG2 expression and the infiltration of M2 macrophages, mast cells, and dendritic cells, as well as a negative correlation with Treg cells and natural killer cells (Figures 5A,B). The data from TIMER database also demonstrated significant positive correlations between PRKG2 expression and the infiltration of CD8+ T cells, macrophages, dendritic cells, and neutrophils (Figure 5C).

### 3.5 PRKG2 inhibits the proliferation and migration of A549 cells *in vitro*

To elucidate the effects of PRKG2 on proliferation, migration, and invasion of A549 cells, we overexpressed PRKG2 in A549 cells *in vitro* (Figure 4B). The MTT proliferation assay indicated that PRKG2 significantly inhibited the growth of A549 cells (Figure 4C). Additionally, another assay showed that elevated PRKG2 expression slowed the wound healing of A549 cells (Figures 4D,E). The Transwell assay revealed that high PRKG2 expression inhibits the migration and invasion of A549 cells (Figure 4E).

## 4 Discussion

Systemic sclerosis (SSc) is an autoimmune disease with one of the highest mortalities (33, 34), leading to substantial suffering and a heavy burden on the lives of affected patients (1). As early as the 1990s, a seminal population-based study by Persson et al. identified a significant increase in cancer incidence among SSc patients (35). Subsequent research from various countries has consistently highlighted an elevated cancer risk in SSc, with a particular emphasis on lung cancer (10, 36–39). Currently, lung cancer is the leading cause of cancer-related death in China, characterized by early metastasis and high mortality (40). Deaths attributable to lung cancer also account for a significant proportion of all-cause mortality in SSc patients (14).

With advances in gene sequencing technology, numerous public databases have been established. Researchers have since developed a range of bioinformatics tools, yielding meaningful findings that have significantly indicated potential research directions. However, variations in sequencing methodologies and standards result in the incomparability of sequencing datasets, posing challenges for analysis. Consequently, drawing on prior research, we applied a novel approach to address these challenges in our study, a method also employed by other investigators (41, 42). The GSE95065 and GSE136043 datasets are standardized transcriptomic profiles from the GEO database, facilitating our analysis of gene expression at the mRNA level. By analyzing differential gene expression of tissues between patients and normal individuals within datasets, we eliminated discrepancies arising from different detection methods and data processing protocols. This approach ensures the comparability of the DEGs from the two datasets.

After ensuring the comparability of results between datasets, we conducted a further search for potential core genes associated with both diseases. We selected varying numbers of DEGs from the datasets with the aim of identifying additional core genes specific to SSc. The linkage analysis of the top DEGs revealed the presence of multiple genes at the core locus for both diseases, with PRKG2 being particularly notable. The intersection of top DEGs also suggested that PRKG2 expression is aberrant in both conditions. Ferroptosis, an iron-dependent programmed cell death, is characterized by the accumulation of lipid peroxides and the peroxidation of unsaturated fatty acids in the cell membrane, resulting in membrane rupture and cell demise (43). Cao et al. demonstrated that ferroptosis in macrophages exacerbates fibrosis in the SSc mouse model (44). Zhang et al. identified ferroptosis and its pro-inflammatory drivers in SSc-ILD at the single-cell transcriptome level (45). Wu et al. observed upregulated expression of ferroptosis-related genes in SSc patients, which are involved in regulating cellular proliferation, differentiation, and migration (46). Also, ferroptosis triggers lung cancer cell death, modulates the tumor microenvironment, impacts metastasis, and alters cell sensitivity to chemotherapeutic agents (47). Considering the impact of ferroptosis on both diseases, we analyzed the correlation between seven shared genes and ferroptosis marker genes, revealing that PRKG2 exhibits the strongest association. Furthermore, analysis of independent validation datasets confirmed that PRKG2 is the most effective gene for distinguishing SSc and LUAD from the normal population, suggesting that PRKG2 is a core gene shared between SSc and lung cancer.

In the GSE95065 and GSE136043 datasets, PRKG2 expressions of SSc and LUAD patients were significantly lower than those of normal individuals, a finding also corroborated by data from the TCGA database. Subsequently, we observed that PRKG2 expressions of LUAD patients across various stages and lymph node metastasis statuses were significantly lower than that of the normal population. LUAD patients with low PRKG2 expression showed significantly shorter median DFS (*p* = 0.028), yet longer median OS (*p* = 0.037). This discrepancy may arise because OS is more susceptible to the influence of various confounding factors, including comorbidities. This suggests that PRKG2 may exert a sustained inhibitory effect on the onset and progression of lung cancer. Building on this hypothesis, we overexpressed PRKG2 in lung cancer cell lines *in vitro* and found that it significantly inhibited cell growth, invasion, and migration, aligning with previous research. Browning et al.’s study suggests that PRKG2 may suppress the proliferation of colon epithelial cells by downregulating Sox9 (48). Additionally, Chen et al. demonstrated that PRKG2 can inhibit the proliferation of various cancer cells, including lung, ovarian, and breast cancer cells, by blocking the MAPK/ERK, PI3K/Akt, Raf/MEK signaling pathways, and EGF/EGFR-related signaling cascades (49–52). Consequently, low expression of PRKG2 facilitates the growth and migration of lung cancer.

The PRKG2 gene, approximately 125 kb in length and comprising 19 exons, is located on chromosome 4 (53). It primarily encodes type II cGMP-dependent protein kinase (cGK II) (54). Reports indicate that robust cGK II signaling is detectable in the lungs of normal mice (55, 56), and PRKG2 is highly expressed in fibroblasts within normal lung tissue (57). As previously mentioned, long-term lung involvement is a notable risk factor for lung cancer development in SSc patients (14). Many researchers also found a correlation between pulmonary fibrosis and the site of lung cancer occurrence, implying that sustained fibrosis may underlie lung cancer development in SSc patients (7, 8, 14). Additionally, some studies propose a link between the carcinogenic mechanism of SSc and pro-fibrotic factors, such as miR-21, miR-199a-3p, miR-199a-5p, and miR-214 (58–61). These molecules, on one hand, enhance the expression of TGF-*β* to promote fibrosis (59), and on the other hand, they may also contribute to tumorigenesis (60). Although the PRKG2 expression levels in pulmonary fibroblasts from SSc-ILD patients compared to those from the normal population in the GSE40839 dataset showed no statistically significant difference, PRKG2 expression levels in pulmonary fibroblasts from SSc-ILD lungs were lower than those in normal lung tissues in both datasets (GSE231693 and GSE40839), with this difference being statistically significant in the GSE231693 dataset. This finding implies that low level of PRKG2 in SSc patients may promote pulmonary fibrosis, leading to the onset and progression of lung cancer.

Immune cell infiltration is recognized to directly modulate the onset and progression of lung cancer, a notion that is widely accepted within the scientific community (62). In our study, the expression level of PRKG2 demonstrated a significant positive correlation with macrophages and dendritic cells within the immune microenvironment of lung cancer. Conversely, it exhibited a negative correlation with Treg cells. These correlations suggest that PRKG2 may play a role in inhibiting lung cancer development through the modulation of these immune cells. This aligns with the findings reported by Kanoh, who concluded that PRKG2 modulates NF-κB activation through PP2A in human cells, thereby influencing innate immunity and inflammation (63). This could elucidate the strong correlation we observed in this study between PRKG2 and innate immune cells. Nevertheless, the current understanding of how PRKG2 mediates immune regulation is limited, necessitating further investigation in future studies.

Our study also has several limitations. Firstly, we analyzed the core shared gene between SSc and LUAD using two datasets and validated our findings with two additional independent datasets, yet potential selection bias may still be present. Secondly, while we examined the effects of PRKG2 on lung cancer cells and the expression of PRKG2 in fibroblasts from lung tissues of SSc-ILD patients, we did not evaluate the effects of PRKG2 in SSc animal models, an aspect we plan to address in future research. Thirdly, although there is a suggested correlation between PRKG2 expression levels and the immune microenvironment of lung cancer, further investigation using immunohistochemical scoring is required due to limited data. Fourthly, due to the absence of demographic and clinical characteristic data, such as SSc subtypes and autoantibodies, from these datasets, we were unable to conduct subgroup analyses to elucidate the impact of these clinical variables on the occurrence of lung cancer in SSc patients. Patients with different SSc subtypes or those carrying various autoantibodies may exhibit distinct gene expression profiles, potentially influencing their development risk of lung cancer. Consequently, the lack of these data may hinder a comprehensive understanding of our study’s findings.

## 5 Conclusion

In summary, this study represents the first to investigate the shared genetic underpinnings between SSc and lung cancer. As a core gene common to both diseases, PRKG2 exerts a significant influence on the proliferation and migration of lung cancer.

## Data Availability

The original contributions presented in the study are included in the article/Supplementary material, further inquiries can be directed to the corresponding author.
