# Supplementary figures and images for: Unveiling the shared genes between systemic sclerosis and lung cancer
Source: Front Med (Lausanne). 2024 Dec 18;11:1431642. doi: 10.3389/fmed.2024.1431642 (PMC11688373; doi:10.3389/fmed.2024.1431642)

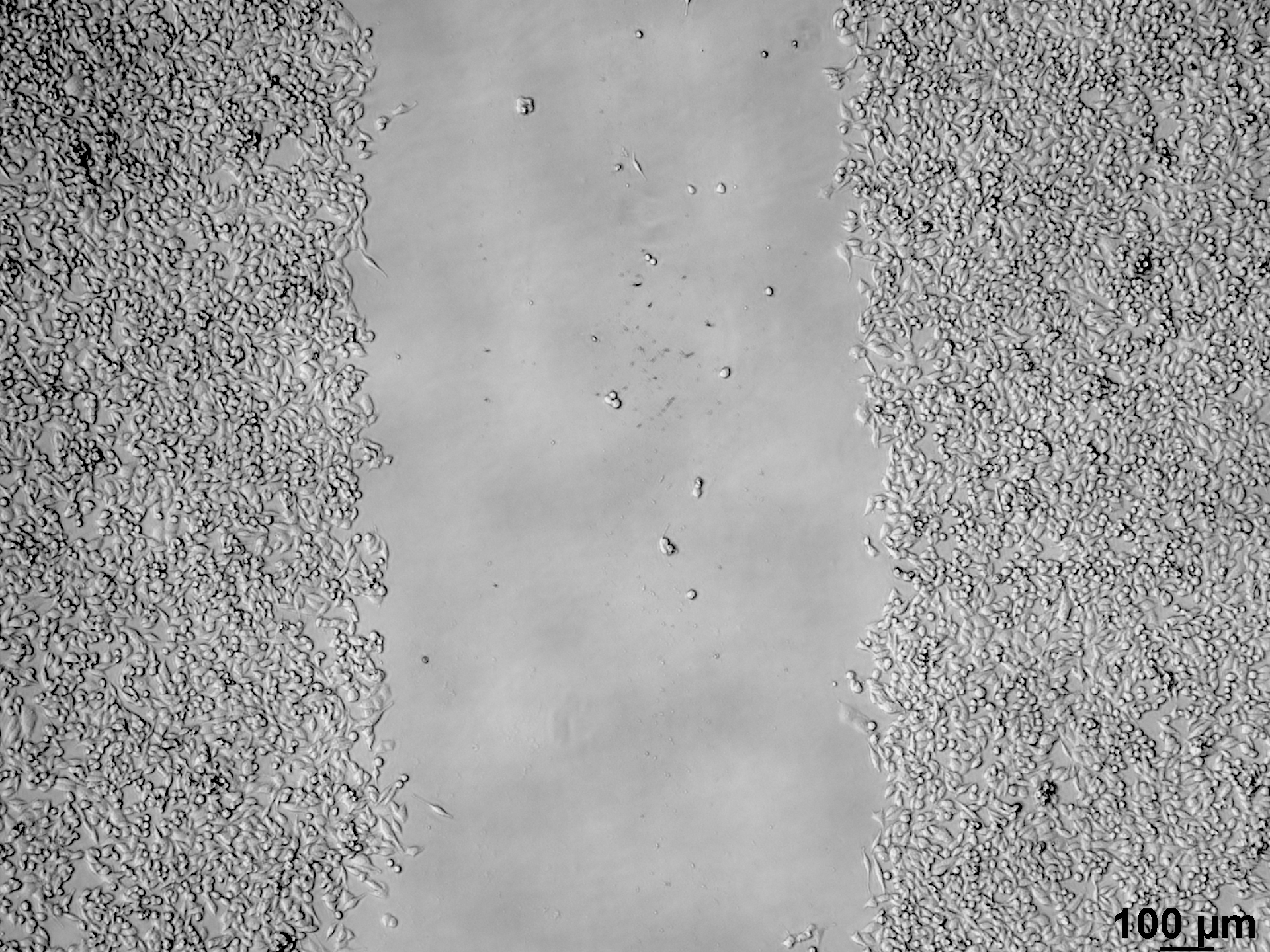

Supplement: Supplementary file 1 [file Image_1.tif]

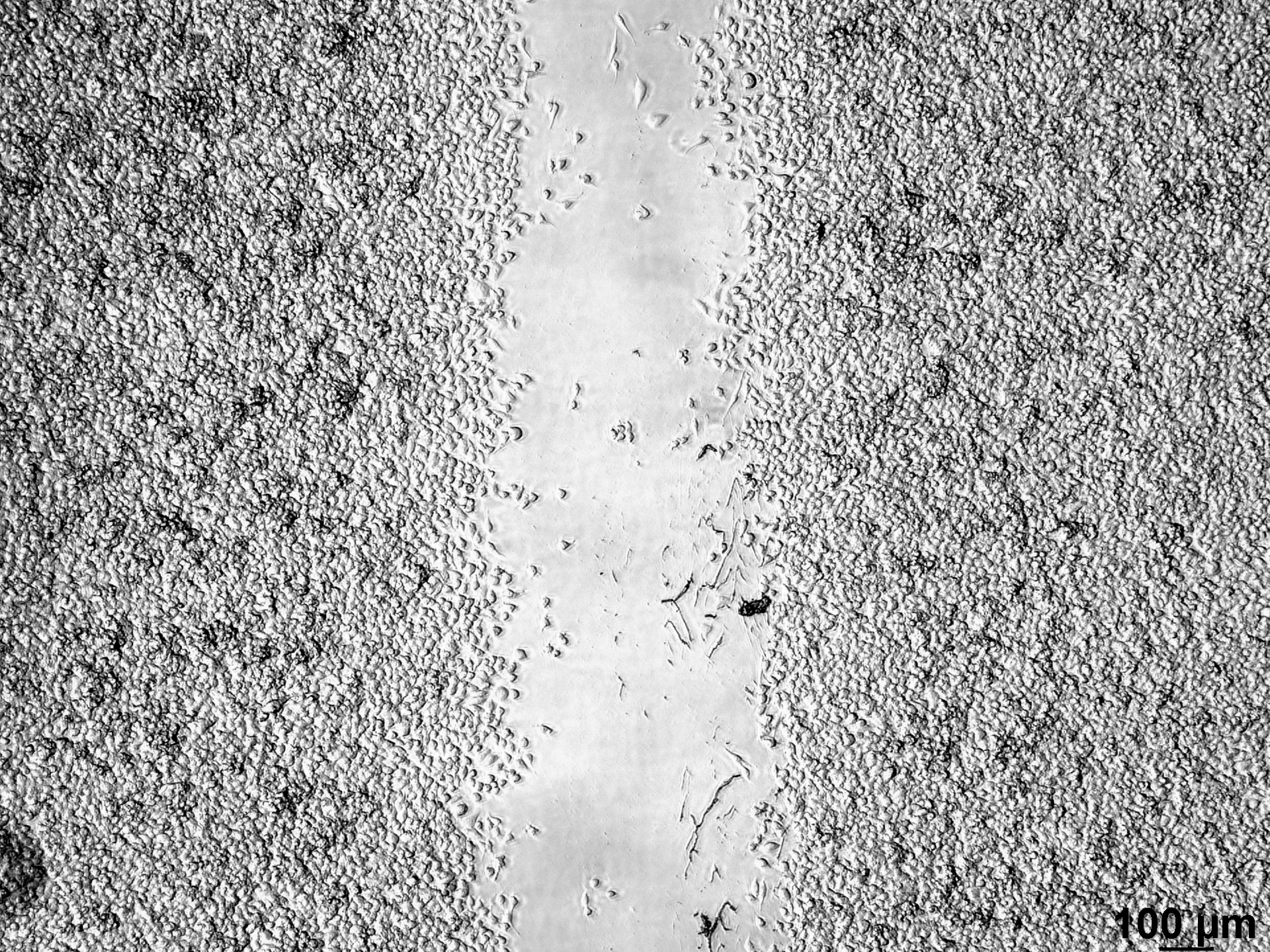

Supplement: Supplementary file 2 [file Image_2.tif]

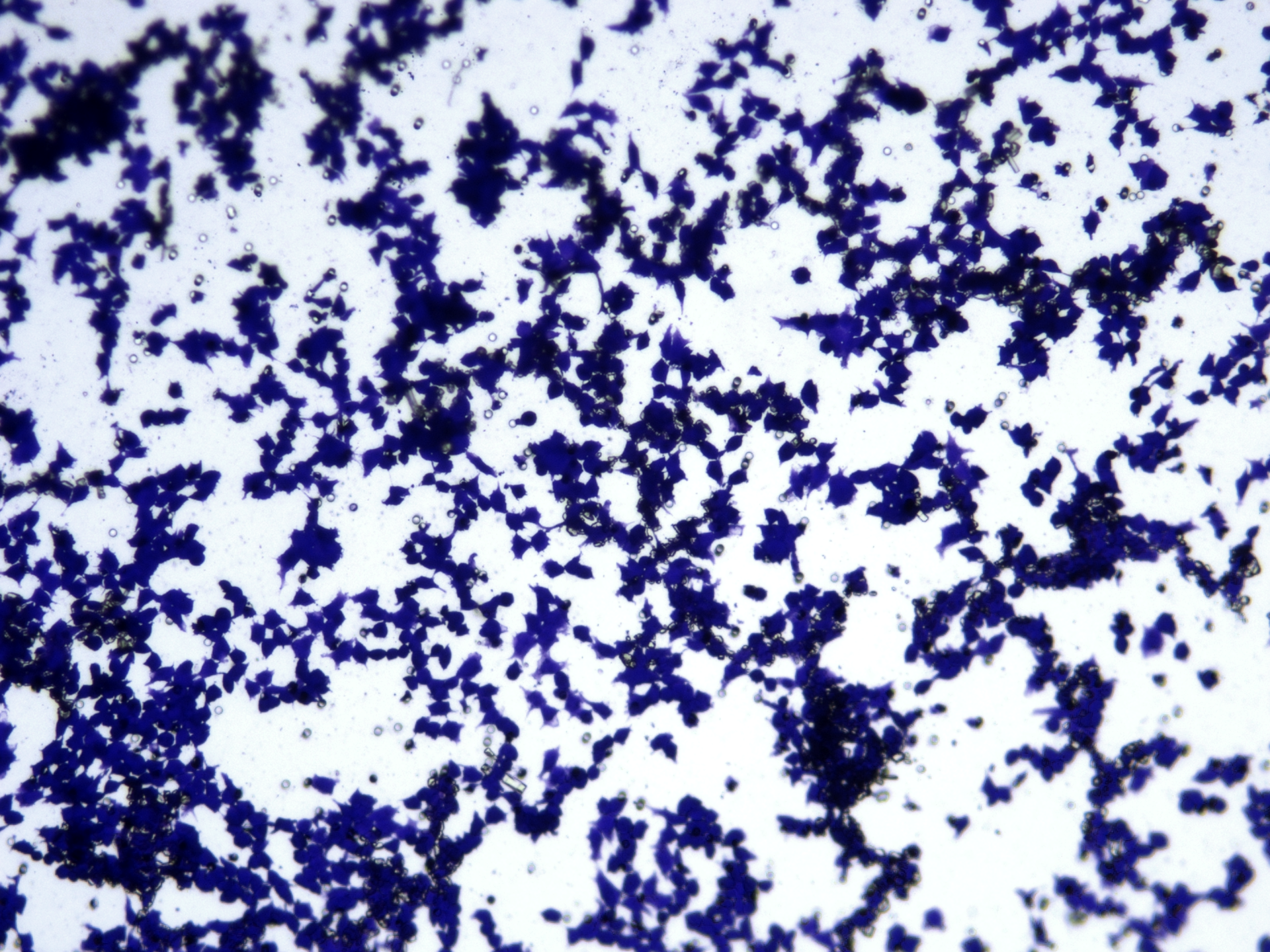

Supplement: Supplementary file 3 [file Image_3.tif]

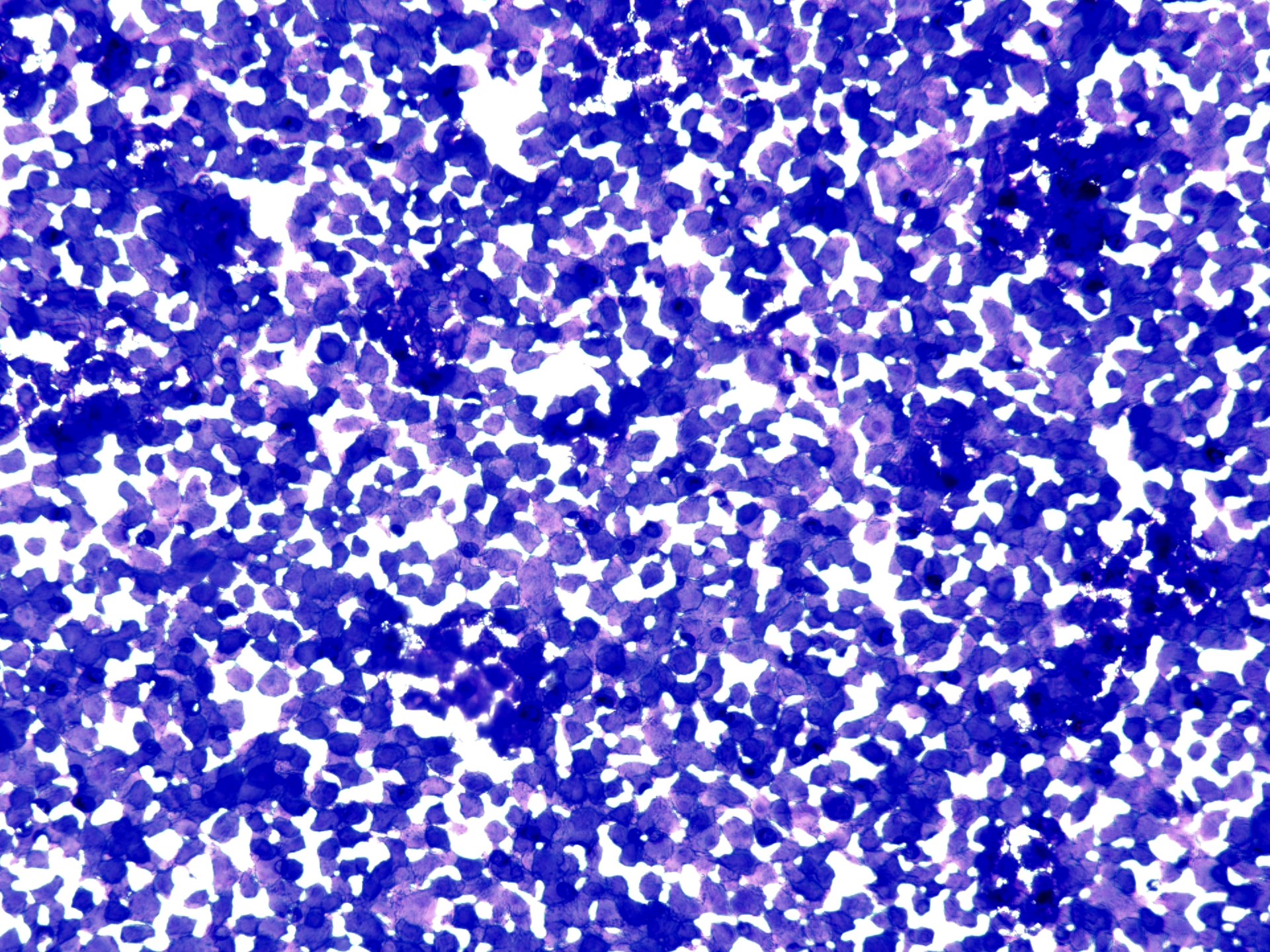

Supplement: Supplementary file 4 [file Image_4.tif]

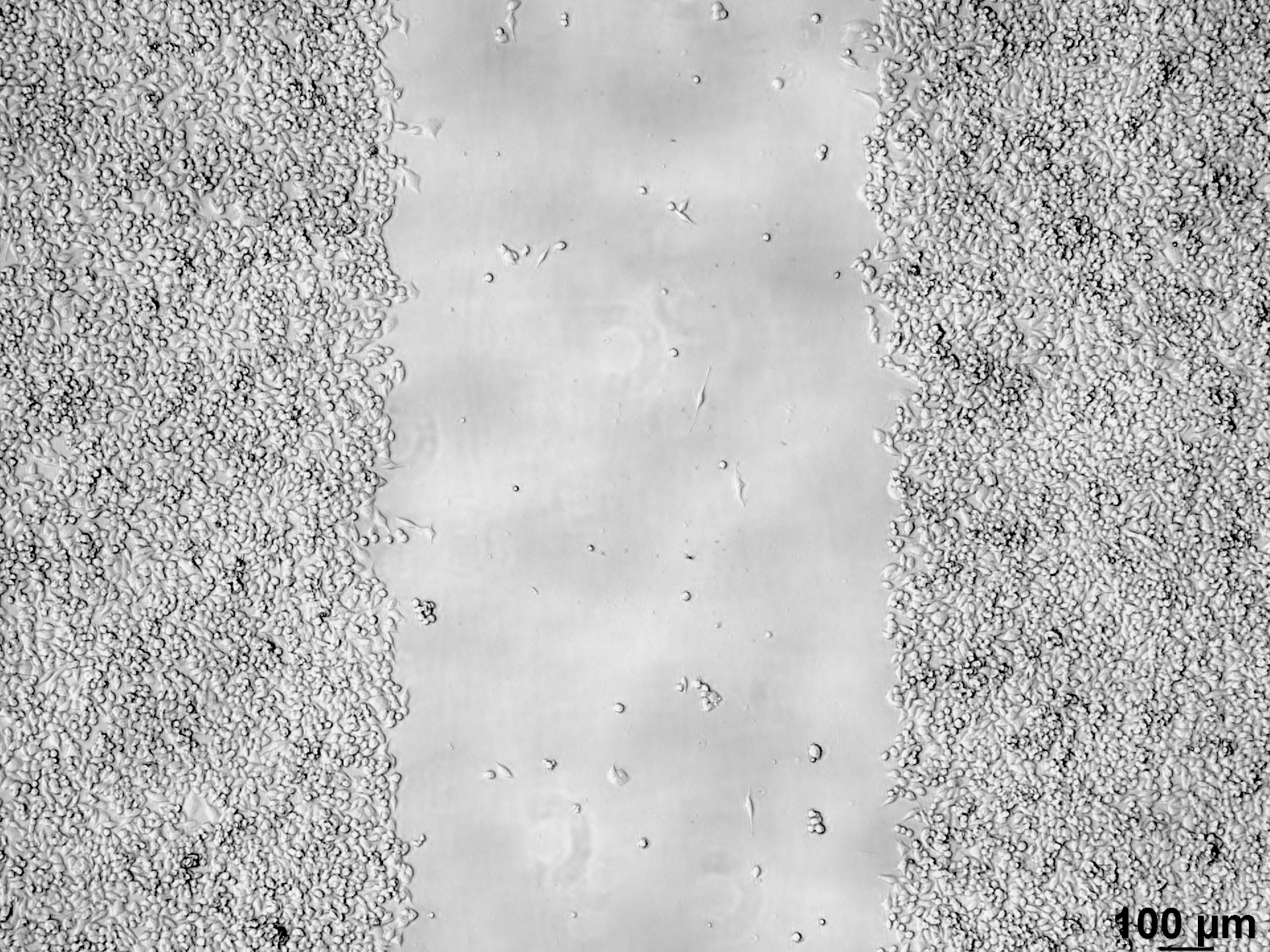

Supplement: Supplementary file 5 [file Image_5.tif]

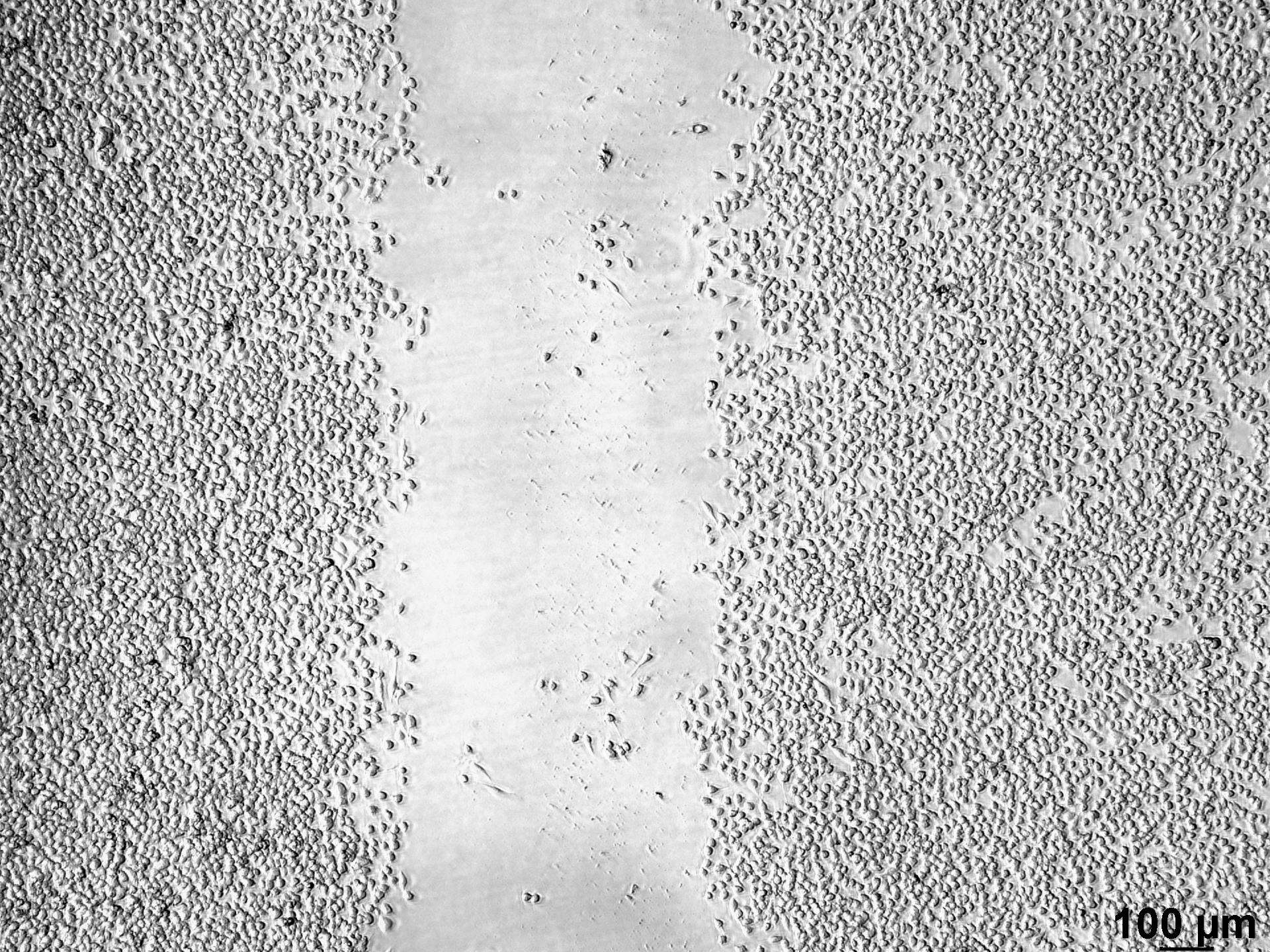

Supplement: Supplementary file 6 [file Image_6.tif]

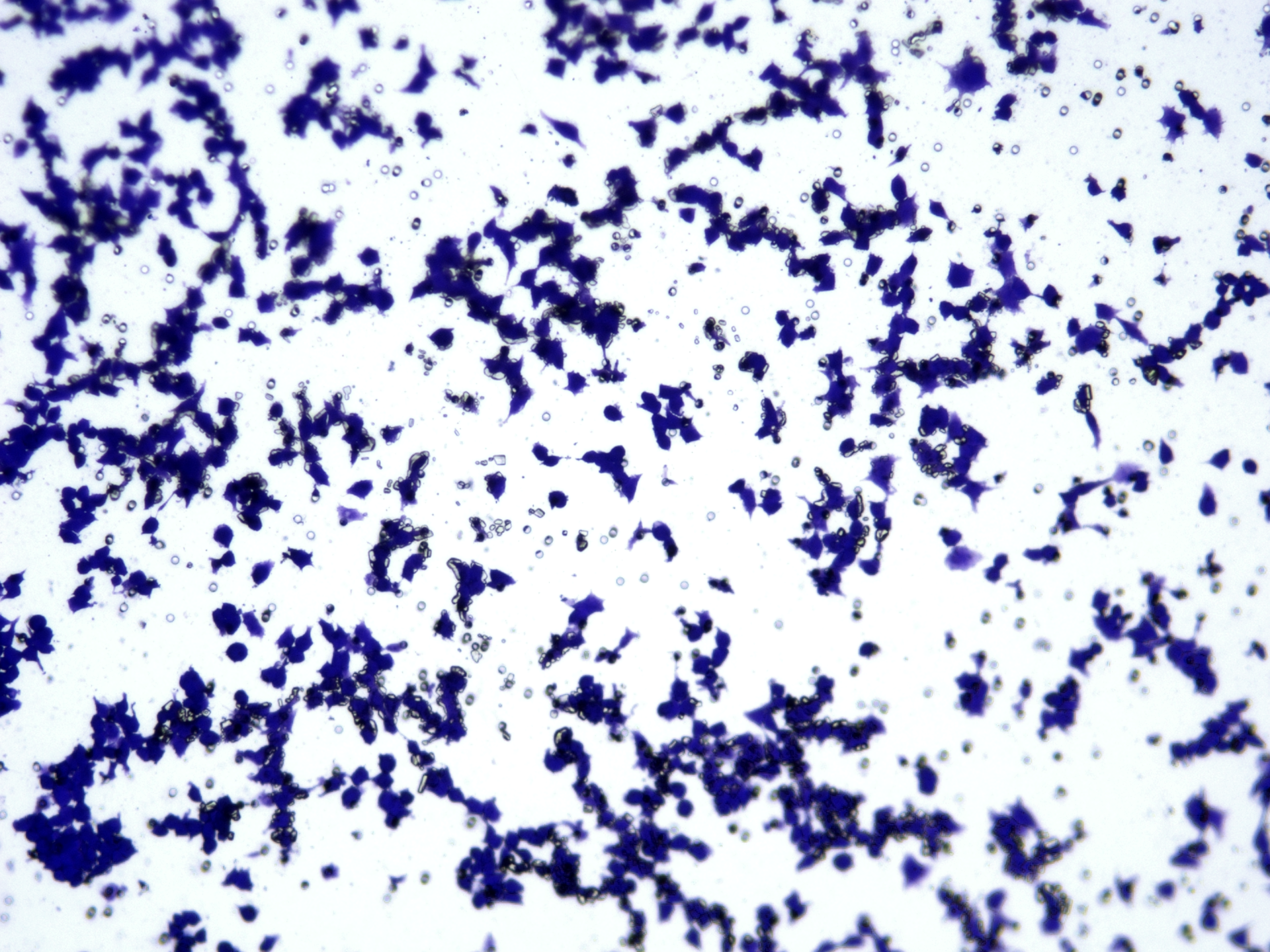

Supplement: Supplementary file 7 [file Image_7.tif]

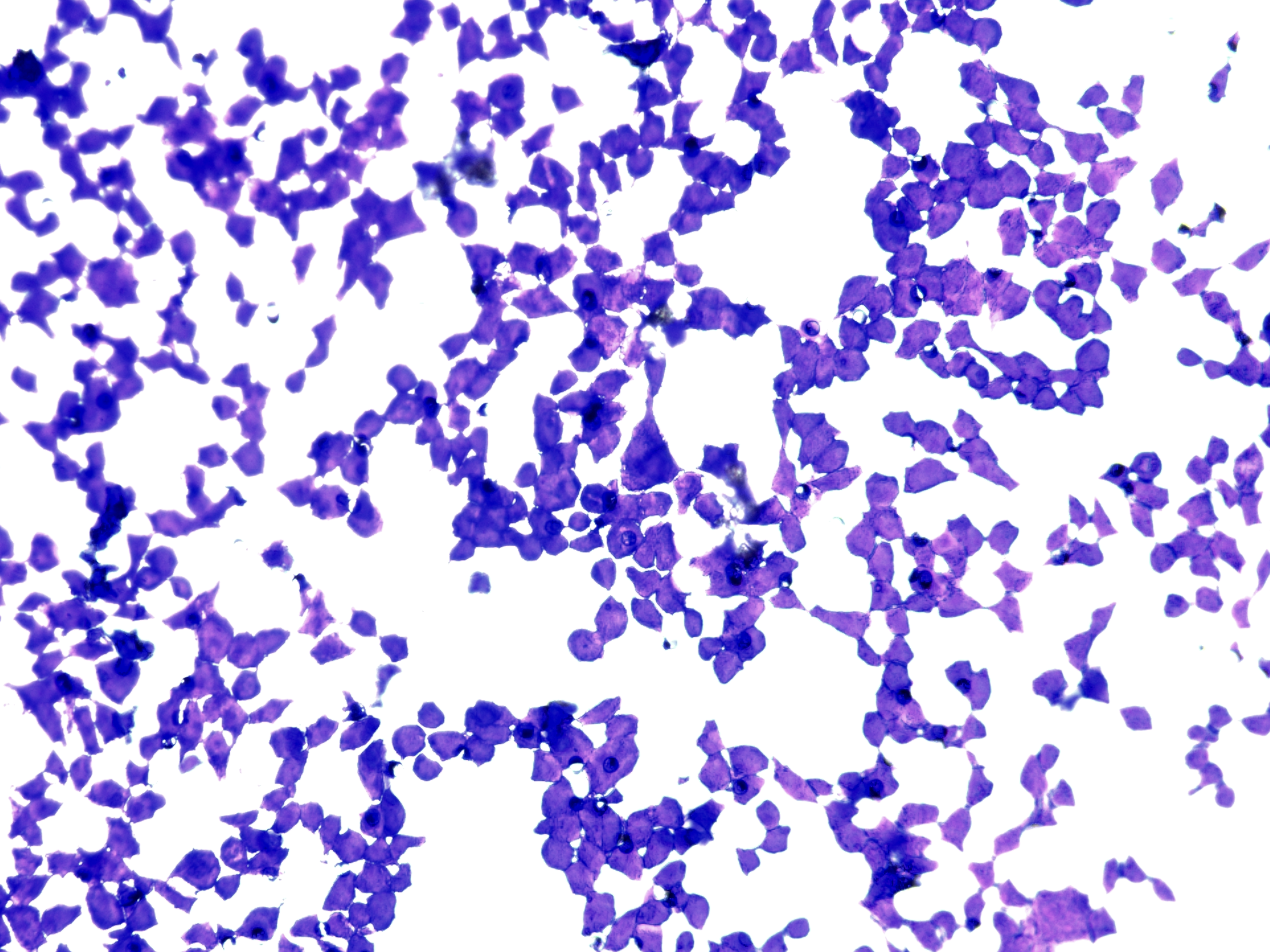

Supplement: Supplementary file 8 [file Image_8.tif]
